# Bioarcheological Perspectives on the Timing of Adolescence in Rural Avar‐Age Austria, 7th–9th Centuries ce

**DOI:** 10.1002/ajpa.70123

**Published:** 2025-09-17

**Authors:** Paul Klostermann, Mary Lewis, Margit Berner, Sabine Eggers, Bendeguz Tobias, Ke Wang, Zuzana Hofmanová, Doris Pany‐Kucera

**Affiliations:** ^1^ Department of Anthropology Natural History Museum Vienna Vienna Austria; ^2^ Vienna Doctoral School of Ecology and Evolution University of Vienna Vienna Austria; ^3^ School of Archaeology, Geography and Environmental Science University of Reading Reading UK; ^4^ Institute for Medieval Research, Austrian Academy of Sciences Vienna Austria; ^5^ Department of Anthropology and Human Genetics, School of Life Sciences Fudan University Shanghai China; ^6^ Department of Archaeogenetics Max‐Planck‐Institute for Evolutionary Anthropology Leipzig Germany; ^7^ Department of Archaeology and Museology Masaryk University Brno Czechia

**Keywords:** bioarcheology, late Avar period, life history, material culture, puberty

## Abstract

**Objectives:**

This study provides insights into adolescent development during the early medieval period in Austria and offers a point of comparison of the timing of sexual maturation relative to the Imperial Roman and the late medieval periods.

**Materials and Methods:**

The timing of adolescent development of 89 individuals in two rural cemeteries from the middle to late Avar period (ca. 650–800 ce) was reconstructed using skeletal and dental indicators. This is the first study to employ genetic sex estimation via ancient DNA on all analyzed adolescents, enabling robust assessment of sex‐specific patterns of growth and development.

**Results:**

Females were on average 1–2 years younger than males at each development stage. Adolescents appear to have developed later during the late Avar period compared to the previous Roman (0.4–2.3 years) and to a lesser extent later than the late medieval period (by up to 1.2 years).

**Discussion:**

These developmental differences may reflect the impact of different living conditions in urban and rural settings as well as underlying genetic variation. While general ages of adolescence were comparable between the early and later medieval groups, the earliest observed age of menarche is 3 years later in the later medieval period than in the Roman group. The timing of the physiological transition is consistent with an increase in grave goods in the early medieval sites. Greater standardization in puberty assessment, age, and biological sex estimation is needed to improve cross‐population comparability of future adolescence studies from different contexts in the past.

## 1 Introduction

Adolescence is the crucial stage in life history, marking the transition from childhood to adulthood. Adolescent development is studied for its plasticity and sensitivity to living environments in anthropology, clinical research, psychology, and more recently, in bioarcheology (Lewis 2022). It reshapes an individual's physiology, psychology, and social roles (Ellison et al. 2012). Nutrition (Villamor and Jansen 2016), disease burden (McDonald et al. 2016), genetic predisposition (Mancini et al. 2022), climate (Canelón and Boland 2020), physiological, and psychological stress (Holdsworth and Appleton 2020), as well as sociocultural conditions (Hermanussen et al. 2012), all have an impact on the onset of puberty and the tempo at which sexual maturation and adult stature are reached. The sex‐specific timing of the adolescent growth phases can be traced through skeletal and dental indicators (Lewis et al. 2016b; Shapland and Lewis 2013, 2014).

Adolescent development has been studied in different periods and regions, revealing the spectrum in the timing of puberty in the past, which has become an additional tool for investigating past living environments (Avery and Lewis 2023; Ham and DeWitte 2022; Lewis 2022). However, we still know little about the full variation of past developmental timing, and data from different geographical and chronological contexts is still needed (Avery and Lewis 2023; Lewis 2022). So far, the largest bioarcheological studies on puberty come from the urban centers of late medieval England (Lewis et al. 2016b), and the Roman Empire in Italy and France (Avery et al. 2023). Moreover, new approaches are needed to address the challenges regarding biological sex estimation in adolescence growing up avar research. Since the timing of sexual maturation differs for females and males, it is critical to establish biological sex, including for the non‐adult remains. Even though sex estimation methods for non‐adult remains have seen promising progress (Sanchez and Hoppa 2023; Stull et al. 2020), estimating biological sex based on morphological skeletal features for non‐adults remains challenging (Lewis 2018). There is a lack of information from Central Europe, with only three individuals from Czechia dating to the Upper Paleolithic studied for adolescent development so far (Lewis et al. 2025). Furthermore, no studies exist for the early medieval period, and there are few studies from rural settlements (Blom et al. 2021).

To address this gap in our knowledge, we analyzed genetically sex‐estimated adolescents from the Avar Period in rural Austria dating to the 7th–9th centuries ce. We reconstructed ages of development stage attainment and the age‐at‐menarche for a rural Avar group and compared them to osteological remains from urban centers of the Roman Empire and late medieval Europe. By comparing urban and rural sexual maturation, we aimed to reveal further information about the impact of environmental factors on the overall health of adolescents in the past, and to explore the biological and social consequences of their development on their communities.

Based on a large comparative study of health indicators which analyzed over 15,119 skeletons from 103 sites spread across Europe from the last two millennia, the early medieval period appears to have been one of the healthiest periods in European history (Steckel et al. 2018). Femoral lengths (*n* = 6739) and enamel hypoplasia prevalences (*n* = 6813) agree that individuals during the early medieval period were taller than late antique and late medieval individuals and suffered relatively fewer growth disruptions and nutritional deficiencies than high and late medieval individuals (Baten et al. 2018; Bereczki et al. 2018; Meinzer et al. 2018; Papathanasiou et al. 2018). These healthier living conditions may be attributed to lower population density and decreased social inequality after the fall of the Western Roman Empire and before the emergence of medieval urbanism (Baten et al. 2018).

### 1.1 Hypothesis

Stress during infancy and childhood, such as poor nutrition and disease, generally delays growth and development (Ham and DeWitte 2022). Therefore, we hypothesize that rural Avar period individuals (7th–9th centuries ce) would show earlier or faster adolescent development than adolescents living in the urban environments during the previous Roman and the following later medieval periods.

## 2 Materials and Methods

### 2.1 Materials

After the Lombards' withdrawal from Pannonia and their migration to Italy, the Avar Khaganate dominated the Carpathian Basin during the early medieval period from 567 to 800 ce (Table 1). The Avar core group of mounted warriors and their families who arrived from the Eurasian Steppe had Northeast Asian and Pontic ancestry, which links them to groups emigrating after the dissolution of the Rouran Empire by the Turks around 550 ce in modern‐day Mongolia (Gnecchi‐Ruscone et al. 2022; Pohl 2018). In the Carpathian Basin, they dominated a multiethnic population whose initially diverse archeological material (late 6th–mid 7th centuries ce) consolidated into a shared archeological culture from the middle Avar period (mid 7th century). By the late Avar period (8th century ce) their society is thought to have become settled, with most of the population living in rural communities practicing subsistence and pastoralist agriculture (Pohl 2018). While there is a lack of archeological evidence for settlements, there is a large number of Avar period cemeteries (Daim et al. 2023). Genetic analysis on the skeletal remains shows a strongly patrilineal and patrilocal social pattern of their communities (Wang et al. 2025). The Avars employed their military expertise, featuring horseback archery, to pressure the neighboring Byzantine Empire to regular tributary payments. However, after their unsuccessful siege of Constantinople in 626 ce, the Avar Empire declined under external and internal pressure until it was destroyed by the Franks under Charlemagne at the beginning of the ninth century (Pohl 2018).

**TABLE 1:** Chronology of the Avar period in early medieval Central Europe (adapted from Daim et al. 2023).

Relative chronological periods	Approximate absolute dating
Early Avar period	~567–650 ce
Middle Avar period	~650–700 ce
Late Avar period	~700–810 ce

**FIGURE 1:**
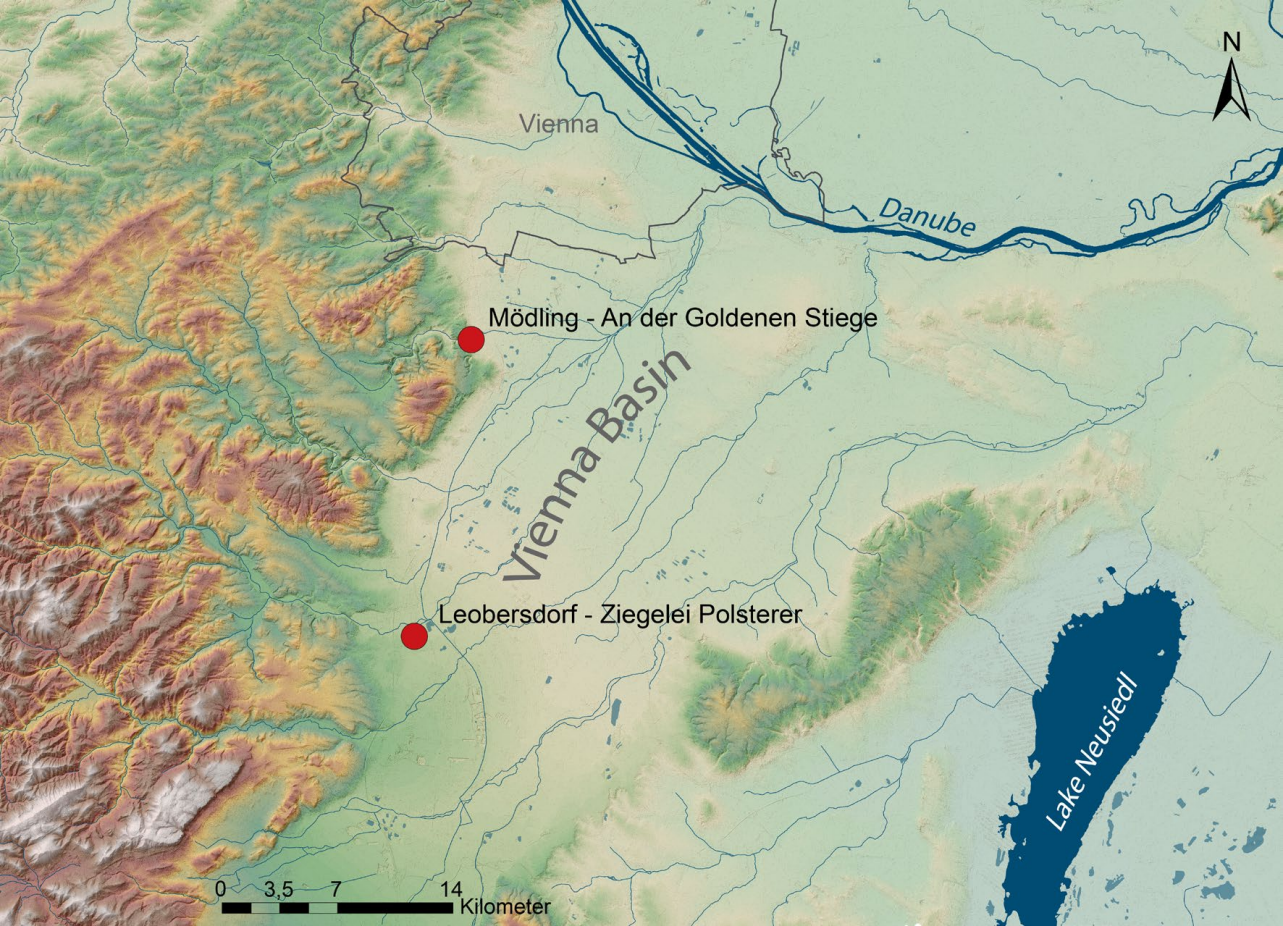
Map with the location of the sites of the Avar cemeteries of Leobersdorf and Mödling, Goldene Stiege in Austria, by B. Tobias.

The cemeteries of Mödling (An der Goldenen Stiege) and Leobersdorf (Ziegelei Polsterer) at the western edge of the Avar Empire are situated in the Vienna Basin south of the Danube River, close to modern‐day Vienna, Austria (Table 2, Figure 1). Archeological evidence coupled with 94 radiocarbon dates and comprehensive genetic analysis show that both burial grounds were used for only 150 years. The occupation of the cemeteries spans six generations, beginning in the middle Avar period (around 650 ce) and was mostly used in the late Avar period until 800 ce (Wang et al. 2025). While the genetic background of the individuals buried there reflects the diversity of the Avar Empire, the graves of both sites contain typical Late Avar ceramics, belt sets, and jewelry. The similar archeological material points to a shared culture and social status between the communities (Daim 1987; Wang et al. 2025), and the geographical proximity of less than 20 km indicates the same topographic and climatic environment. Hence, the burials from the two contemporaneous sites were combined to increase the sample size. In total, 89 individuals between the ages of 8 and 30 years with genetic sex and sufficient maturity indicators could be included. Destructive sampling and analysis of human remains were undertaken with the approval of the Natural History Museum Vienna and following the museum's ethical sampling protocols. There are no claims of descendant communities and there was no community involvement.

**TABLE 2:** Skeletal collections analyzed for this study.

Site	No. of individuals with genetic sex (8–30 years)	No. of individuals with adolescence stage
Mödling, Goldene Stiege	96	63
Leobersdorf, Ziegelei Polsterer	47	26
Total	143	89

**FIGURE 2:**
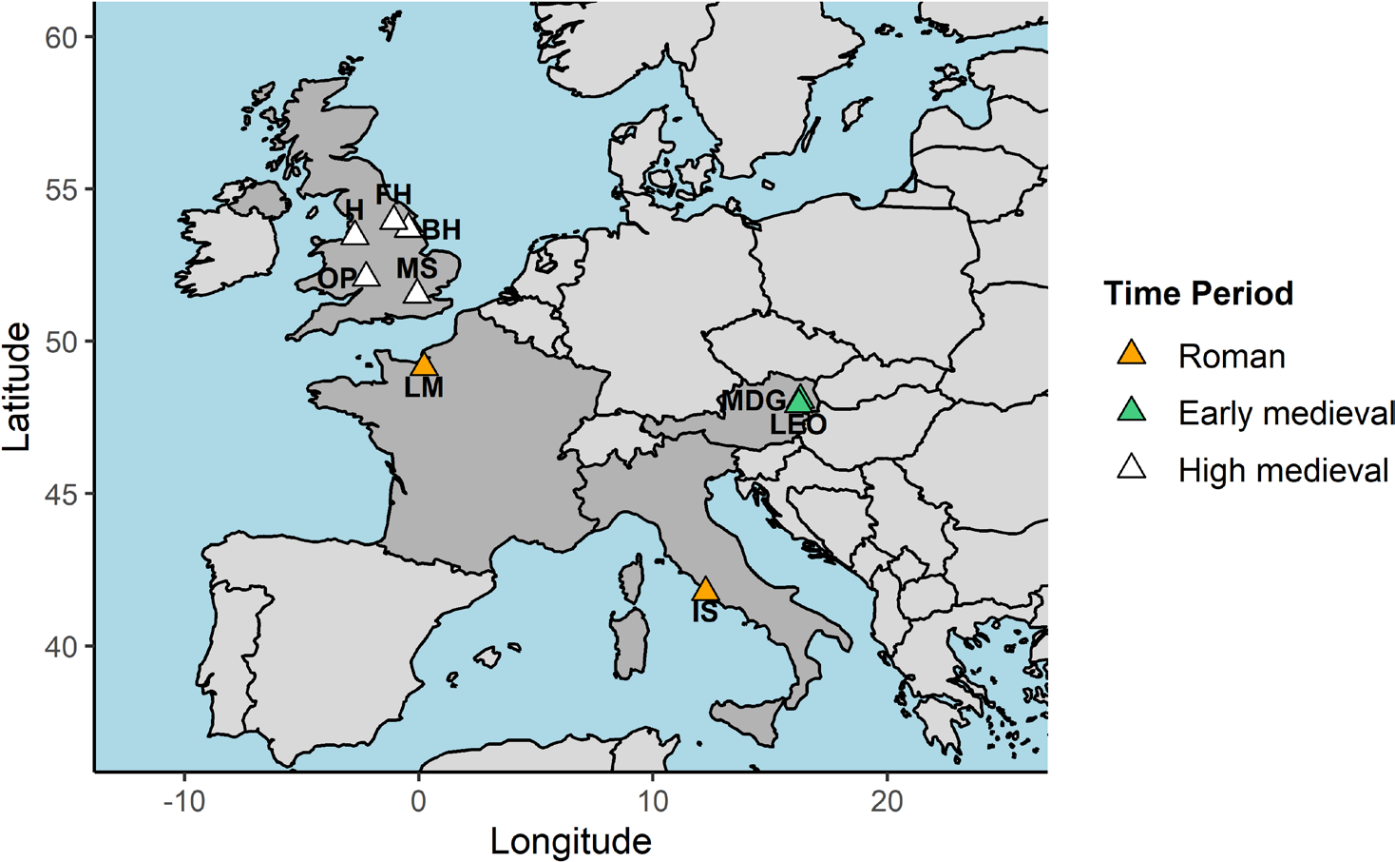
Map of Europe showing all sites used in this study. Green: Early Medieval Period sites (EMP) newly analyzed for this study, MDG: Mödling, Goldene Stiege, LEO: Leobersdorf. White: High/Late Medieval Period (Lewis et al. 2016b) (HMP), MS: St. Mary Spital, BH: Barton‐on‐Humber, FH: Fishergate House, H: St. Helens, OP: St. Oswald's Priory. Orange: Roman sites (Avery et al. 2023). IS: Isola Sacra, LM: Lisiseux‐Michelet.

The early medieval skeletons analyzed in this study were compared to previously published adolescents with an assigned puberty stage and from the Roman Empire in France and Italy (Avery et al. 2023) and to late medieval adolescents from England (Lewis et al. 2016b) (Table 3, Figure 2). These data sets constitute the only large bioarcheology studies on pubertal timing to date. They also include biological sex estimation, which enables a sex‐specific comparison.

**TABLE 3:** Published adolescent samples used as comparison in this study.

Period	Dates	Sites	Context	No. of adolescents	No. of adolescents with puberty stage	References
Roman	1–4th cent. ce	Isola Sacra, IT	Urban, middle status	127	127	Avery et al. 2023
	4–5th cent. ce	Lisieux‐Michelet, FR	Urban, middle status	137	137	
Late medieval	10–17th cent. ce	St. Mary Spitals, London, UK	Urban, low status, probably inadequate diet	704	759	Lewis et al. 2016b
	10–17th cent. ce	York, several, UK	Urban, low status	90		
	10–17th cent. ce	Barton upon Humber, UK	Urban, high status	200		

### 2.2 Methods

#### 2.2.1 Biological Sex Estimation

This study only includes non‐adults whose biological sex was estimated by ancient DNA (aDNA) analysis, primarily from petrous bones and teeth (Wang et al. 2025). Analyses were performed at the Department of Archaeogenetics, Max Planck Institute, according to established protocols (Wang et al. 2025). In this study, sex estimation is used to account for the variation in growth trajectories associated with biological sex (Bogin 2020). Chromosomal sex estimated using aDNA provides a robust proxy for biological sex than is possible via morphological sex estimation from non‐adult remains despite limitations such as contamination, degradation, and chromosomal variation (Brown and Brown 2011, 118–162). This binary framework does not reflect all biological variations of the sex spectrum (Anastasiadou et al. 2024), but supports comparability with the medical and bioarcheological literature on adolescent development (Ham and DeWitte 2022, 2–3; Lewis 2022, 3).

#### 2.2.2 Age‐at‐Death Estimation

Age‐at‐death was estimated using dental mineralization (8.0–17.9 years), a combination of dental mineralization and epiphyseal fusion (18.0–23.9 years), or epiphyseal fusion when dental development was complete (24–30 years). Dental age was estimated before knowing genetic sex using the mineralization tables from the London Atlas (AlQahtani et al. 2010). This method was chosen as it allows a mean age estimation from both maxillary and mandibular teeth and is reliable for diverse living populations (Jacometti et al. 2023). Radiographs were taken in cases where tooth roots were not observable. To enhance the resolution of adolescent growth endpoints, the young adult age categories were narrower than those conventionally used. After dental mineralization was complete, individuals were placed in age categories of 18.0–23.9, 24.0–25.9, and 26.0–30.0 years (Table S1). To assess age independently of pubertal status, any indicators, which were used in puberty assessment, such as mandibular canines and the epiphyses described below, were excluded from age estimation. This was done to avoid circularity in the analysis of pubertal timing (Lewis et al. 2016a, 2016b). Because some studies reported the onset of puberty before 10 years and incomplete development up to 25 years (Blom et al. 2021, Lewis et al. 2016b), the common age range from 10 to 25 years was extended to 8–30 years to capture the full range of adolescence.

#### 2.2.3 Assessment of Adolescent Development

The methods by Shapland and Lewis (2013, 2014) and Lewis et al. (2016b) were used to estimate where along the adolescent growth spurt an individual was at the time of their death. Individuals were placed in one of seven stages: pre‐puberty, onset, acceleration, peak height velocity (PHV), deceleration, maturation, and completion. A development stage was only assigned to individuals with at least three indicators (Table 4) which agreed on one stage to ensure reliability.

**TABLE 4:** Adolescence stage indicators used in this study (modified from Lewis et al. 2016 and Falys and Lewis 2020).

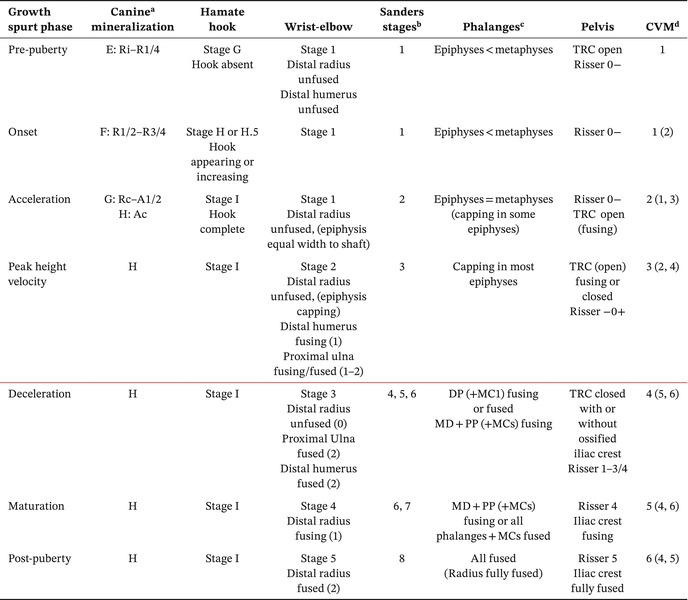

*Note:* The red line shows when menarche is usually achieved.

Abbreviations: CVM, cervical vertebrae maturation; DP, distal phalanges; MC, metacarpal; MD, middle phalanges; PP, proximal phalanges; TRC, triradiate cartilage.

^a^ Canine mineralization stages in Demirjian stages E–H with corresponding stages after Moorrees et al. (1963).

^b^ Sanders et al. (2008).

^c^ Any present phalanx was scored as a proxy for the rest of the phalanges of the same type (Hewitt 1963).

^d^ CVM can be off by one stage and still agree (Avery and Lewis 2023). Stages 4–6 indicate deceleration to completion stage (McNamara and Franchi 2018).

The variability of cervical vertebrae maturation (CVM) was accounted for by allowing a one‐stage difference between indicators following Avery and Lewis (2023). Additionally, CVM phases 4–6 were all regarded to agree with deceleration, maturation, and completion phases following the clinical guide by McNamara and Franchi (2018).

Fusion of proximal, intermediate, and distal phalanges, and metacarpals, was recorded separately and assessed following Shapland and Lewis (2013) with additional insights from Sanders et al. (2008) (Table 4). Developed from radiographs of females with idiopathic scoliosis, the Sanders method combines earlier approaches (Greulich and Pyle 1959; Tanner et al. 2001) and shows a strong correlation with the pubertal growth spurt (Sitoula et al. 2015) allowing for greater variability in stage assessment. Iliac crest ossification and fusion of the ilium, ischium, and pubis at the triradiate cartilage were recorded following the Risser scale (Negrini et al. 2015) (Table 4). Although developed for adolescents with idiopathic scoliosis, who might differ from the general population, Risser scores correlate with the pubertal growth spurt (e.g., Charles et al. 2006) and thus serve as proxies for growth phases in other contexts. An unfused triradiate cartilage typically persists until PHV, and it usually fuses at the end of this phase (Neal et al. 2018; Sanders et al. 2007). An ossified but unfused iliac crest (Risser 1–3/4) indicates deceleration, while a fusing crest indicates maturation phase and complete fusion marks the end of the growth spurt (Lewis et al. 2016b). Often in archeological contexts, the iliac crest could be missing for taphonomic reasons. If no iliac crest is found and the triradiate cartilage is unfused, the individual is likely in onset, acceleration, or PHV, whereas a fused triradiate cartilage without an iliac crest suggests deceleration or PHV.

#### 2.2.4 Assessment of Menarche Status

Menarche typically occurs shortly after the distal hand phalanges start fusing (Frisancho et al. 1969), and just before ossification of the iliac crest (Risser 1) at the beginning of the deceleration phase (Buehl and Pyle 1942). Accordingly, fusing distal phalanges were considered indicators that menarche was occurring around the time of death (Buehl and Pyle 1942; Frisancho et al. 1969), while an ossified but unfused iliac crest indicated that menarche had recently occurred (Buehl and Pyle 1942). To investigate shifts in menarcheal age between populations, females were separated into pre‐ and post‐menarche categories based on osteological indicators following DeWitte and Lewis (2020) (Table 5).

**TABLE 5:** Indicators for menarche status (after DeWitte and Lewis 2020; Lewis 2016).

Pre‐menarche	Post‐menarche
Unfused hand phalanges	Fusing^a^/fused hand phalanges
Unfused triradiate cartilage (Risser 0–)	Risser 1–5
Incomplete hamate hook	—
Developing canine root	—
Unfused proximal ulna	Distal radius fusing
Unfused distal humerus	—
CVM 3 or less	CVM 4 or more

^a^ The one female with fusing distal phalanges who likely died around the time of menarche was categorized with the post‐menarche group for this analysis.

#### 2.2.5 Statistical Testing

Descriptive and inferential statistics were calculated using Microsoft Excel and R Version 4.4.0 with R Studio 2024.04.2. Owing to non‐normal data distribution, non‐parametric tests, that is, the Mann–Whitney *U*‐test and the Kruskalis–Wallis test, were applied to assess differences in mean ages between groups. Statistical significance was set at *p* < 0.05, with *p*‐values adjusted using the Bonferroni–Holm method to correct for alpha error accumulation. Effect sizes were calculated to estimate the strength of differences: Hedge's *G* was used for the Mann–Whitney *U*‐test, and epsilon squared (*ɛ* ^2^) was used for the Kruskalis–Wallis test, with interpretation according to Cohen (1988) (Table 6).

**TABLE 6:** Interpretation of effect sizes.

Interpretation	Effect size	
	Hedge's *G*	Epsilon^2^
Negligible	< 0.2	—
Small	0.2–0.4	< 0.08
Moderate	0.5–0.7	0.08–0.26
Strong	> 0.7	≥ 0.26

## 3 Results

### 3.1 Preservation, Age, and Sex Estimation

**FIGURE 3:**
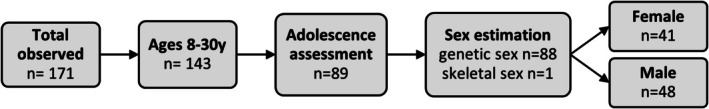
Breakdown of sample size of adolescents from the early medieval period in the Vienna basin.

**FIGURE 4:**
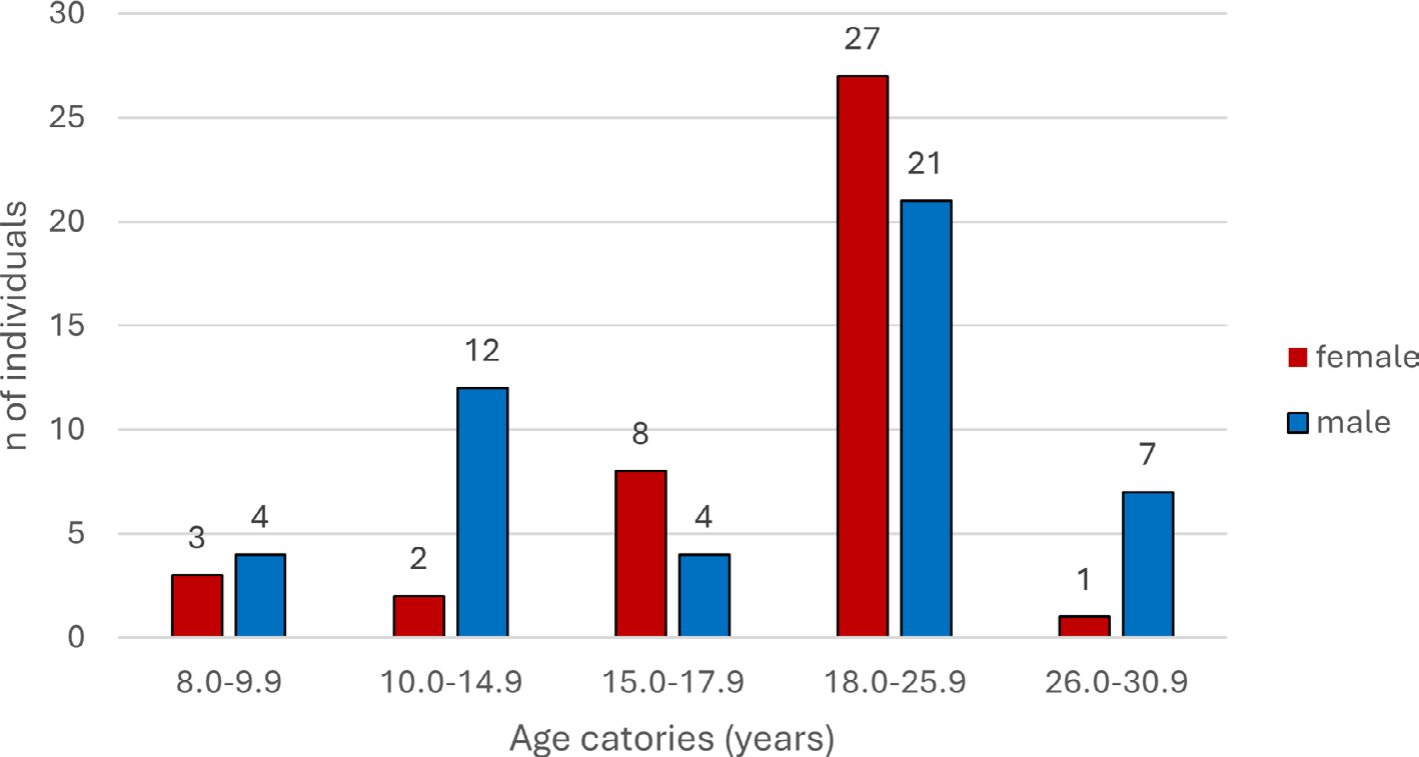
Age and sex distribution of the 89 genetically sexed early medieval adolescents with an adolescent development stage. From a modern biological and psychological perspective, adolescence can be broken down into early (10–14 years) middle (15–17 years), and late (18–25 years) phases (Lewis 2022).

Of the total 171 individuals analyzed, 143 were aged between 8 and 30 years (Figure 3). Genetic sex estimation was successful for 88 of the 89 individuals for whom an adolescence stage could be assessed (Wang et al. 2025) and failed in one young adult individual for whom standard morphological sex estimation was applied (Buikstra and Ubelaker 1994). The final study sample comprised 89 individuals (41 females; 48 males) for whom an age‐at‐death and adolescent developmental stage as well as the biological sex could be assessed (Table 7, Figure 4). The sample size was affected by poor preservation at both sites, with smaller bone elements, such as the hand phalanges, often missing. When scoring puberty stage, the mineralization of the mandibular canine was the development indicator that could be scored most and the hamulus of the hamate bone the least often (Table 8). Furthermore, likely due to low mortality rates among adolescents (Avery and Lewis 2023; Lewis 2007) the age groups between 8 and 18 years are naturally represented by fewer individuals. The age‐at‐death of all non‐adults was estimated via dental age; 18 out of 27 individuals of 18–23‐year‐olds and all individuals above that age were estimated using epiphyseal fusion.

**TABLE 7:** Age and sex distribution of early medieval individuals with puberty assessment.

Site	Age category (years)	Biological sex		Total
		Female	Male	
Leobersdorf	8.0–9.9	0	0	0
Early adolescence	10.0–14.9	0	2	2
Mid adolescence	15.0–17.9	2	1	3
Late adolescence	18.0–25.9	13	8	21
	26.0–30.9	0	0	0
	Total	15	11	26
Mödling, Goldene Stiege	8.0–9.9	3	4	7
Early adolescence	10.0–14.9	2	10	12
Mid adolescence	15.0–17.9	6	3	9
Late adolescence	18.0–25.9	14	13	27
	26.0–30.9	1	7	8
	Total	26	37	63
Grand total		41	48	89

**TABLE 8:** Preservation of used adolescent development indicators.

Development indicator	Observable *n* (total *n* = 143)
Mandibular canine	95 (66.4%)
Elbow–wrist	80 (55.9%)
Hand phalanges	78 (54.5%)
Pelvis	74 (51.7%)
CVM	68 (47.6%)
Hamate hook	17 (11.9%)

### 3.2 Pubertal Timing in Early Medieval Adolescents

**FIGURE 5:**
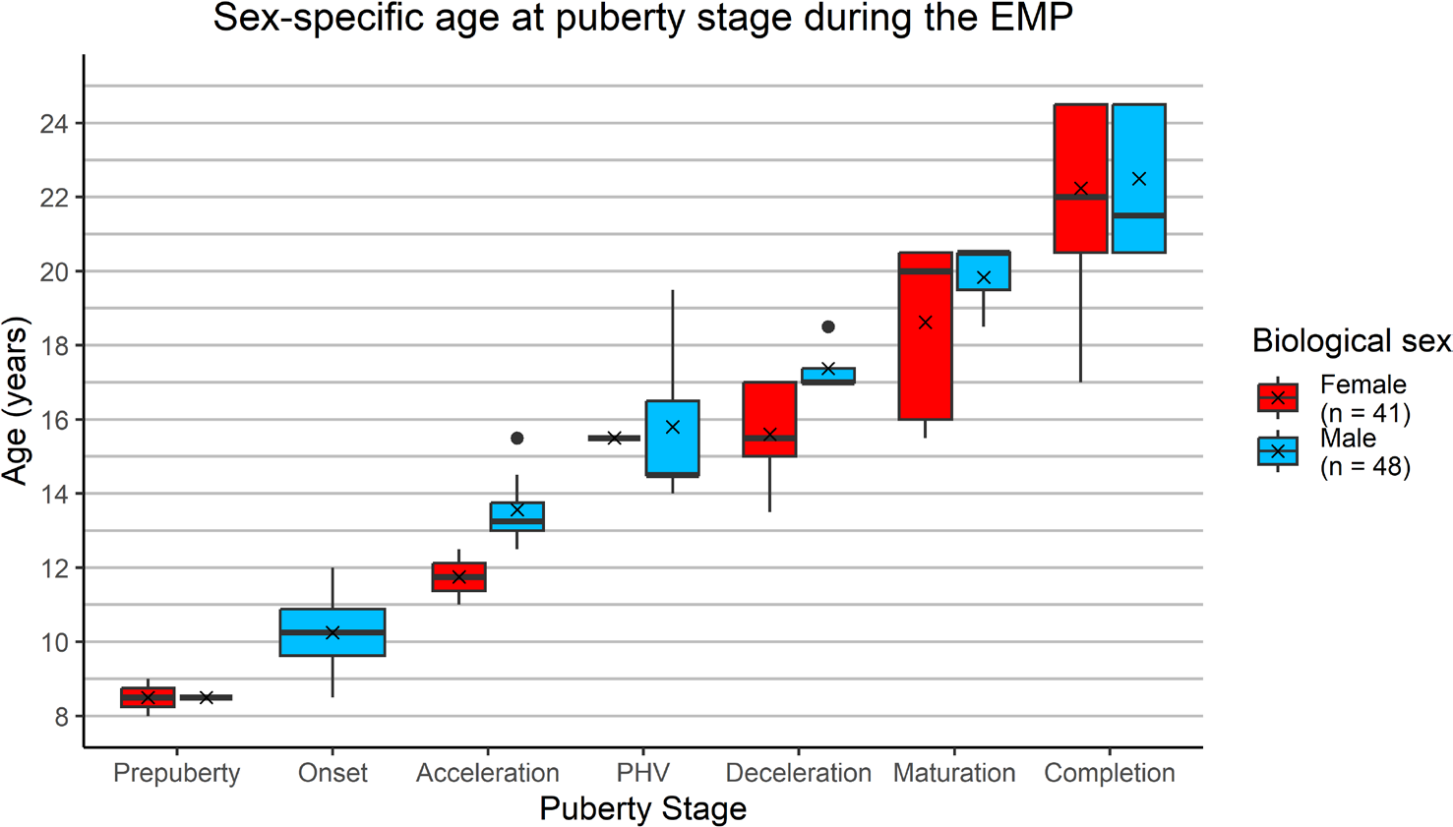
Mean ages of females and males during the Early Medieval Period (ages 8–25) in the Vienna Basin, Austria. Biological sex was estimated via aDNA.

Eighty‐nine individuals had at least three development indicators preserved that agreed on a development stage (Table 4). Females were on average 0.3–1.8 years younger compared to males during each adolescence stage (Figure 5, Table 9). In the 18–23 year age category, five out of 14 females and five out of 12 males had not finished their growth at this age. All individuals had finished growth by the age of 23 years. However, while those females were all in the maturation stage, one male was in deceleration and another was still at PHV aged 18–23 years. When the sexes were combined, puberty started on average at 10.3 years, PHV occurred around 15.6 years, and development was complete by 22.3 years (Table 9).

**TABLE 9:** Comparison of sex‐specific mean age and age range at puberty stages of Roman, early and late medieval populations.

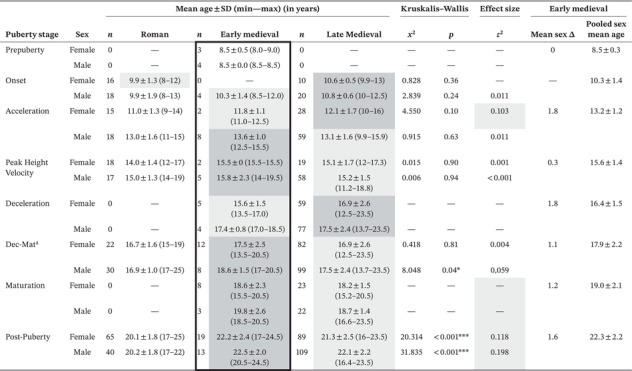

*Note:* Highest mean age in row in dark gray, second highest mean age in light gray, lowest mean age in white. Effect sizes in gray indicate moderate effects. Thick border marks early medieval individuals with sex estimation based on genetics. *marks significant results with *p* < 0.5. ***marks significant results with *p* < 0.001.

^a^Dec‐Mat combines deceleration and maturation stages in early and late medieval individuals.

#### 3.2.1 Menarche in Early Medieval Females

**FIGURE 6:**
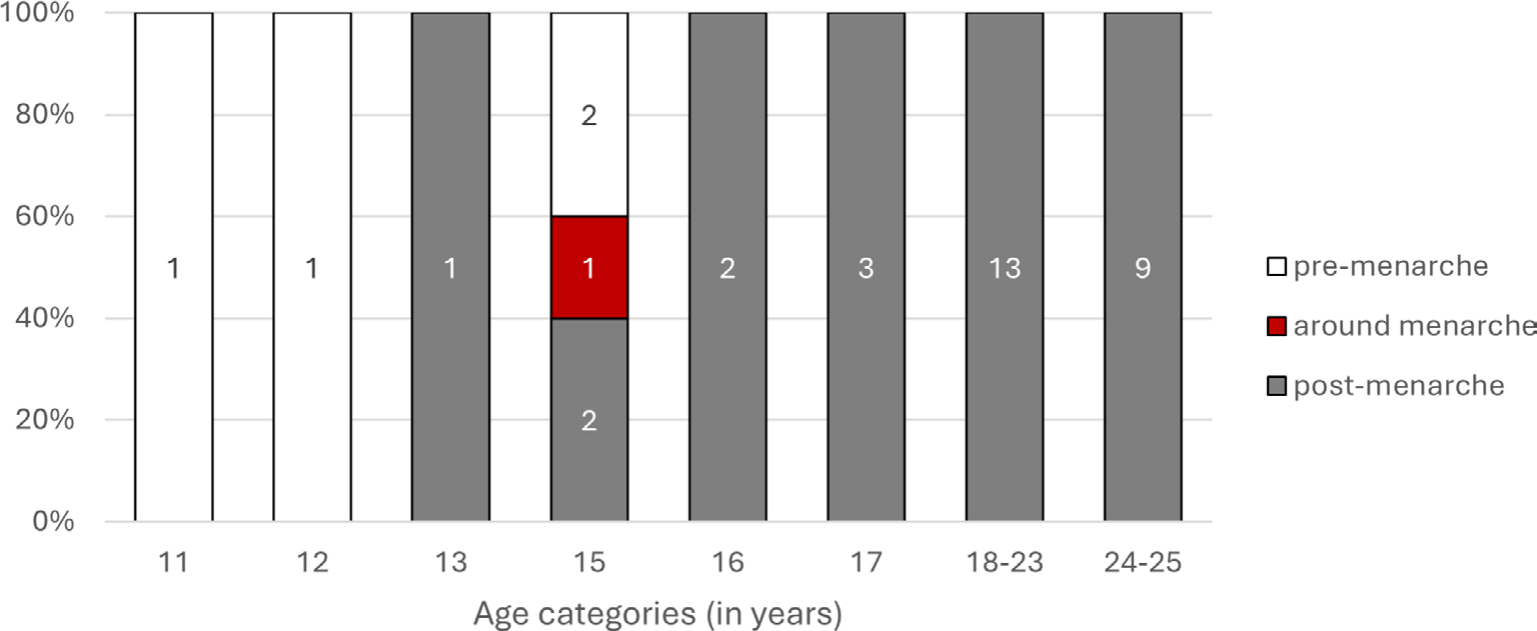
Menarche status at age for early medieval females.

Menarcheal status could be assessed in 15 females between the ages of 10 and 18 years. Only one 15‐year‐old female from Leobersdorf had preserved distal phalanges that were fusing, indicating that she died around the time of menarche. No females had an ossified but unfused iliac crest. However, two females with a mean age of 14 and 15.5 years from Mödling had a fusing triradiate cartilage, which indicates that they were at the end of PHV just before menarche usually occurs. Additionally, six females from the ages of 13.5–17.0 years had a fused triradiate cartilage and an unfused and unrecovered iliac crest, suggesting that they died around the time of menarche, right before or during the deceleration phase. The oldest females who were likely pre‐menarcheal had a mean age of 15.5 years, and the youngest female to have passed menarche was 13.5 years old (Figure 6).

#### 3.2.2 Comparison to Roman and Late Medieval Populations

**FIGURE 7:**
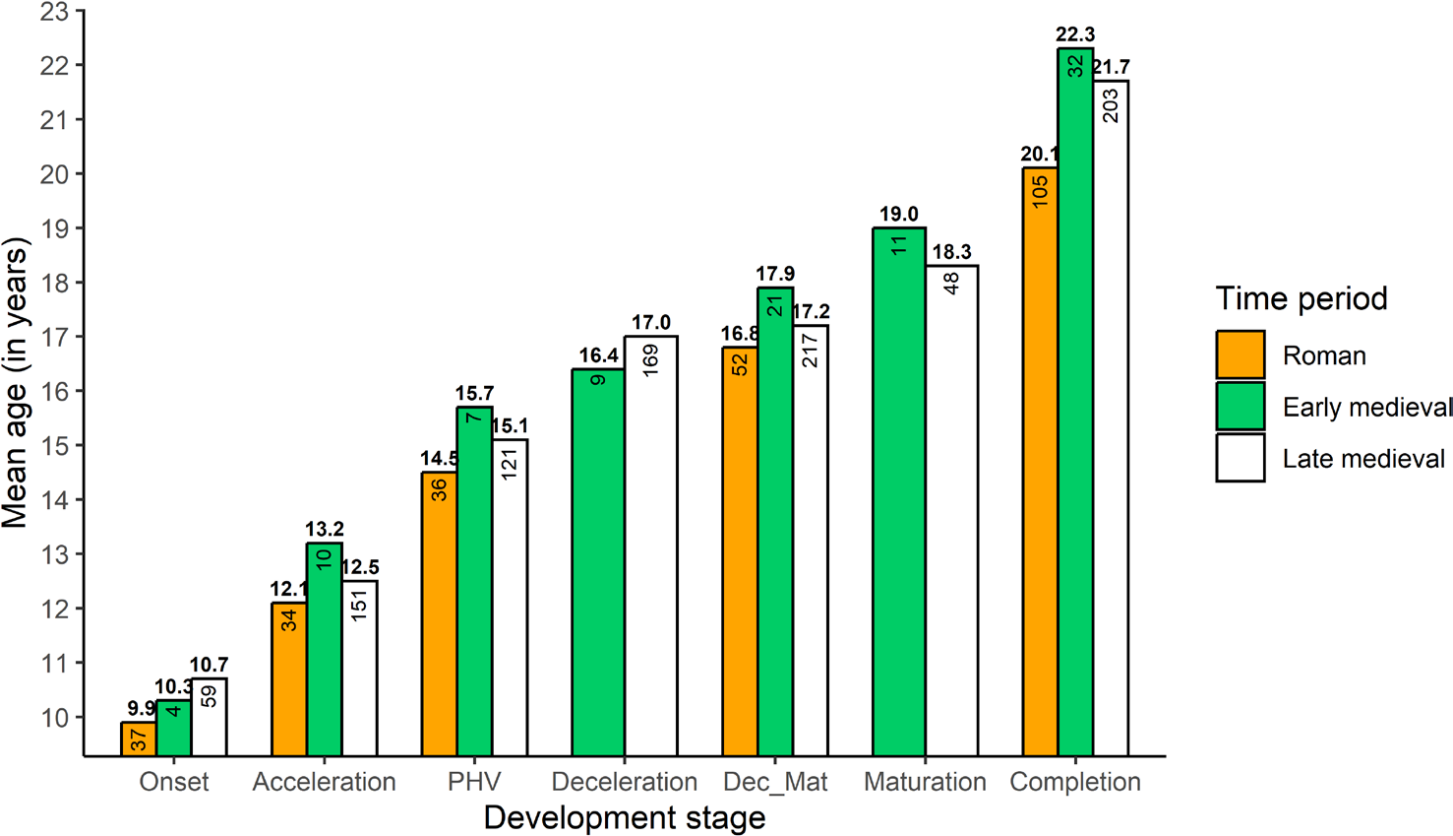
Comparison of mean age at puberty stage. For a comparison with the Roman period, deceleration and maturation were combined into one stage (Dec‐Mat).

**FIGURE 8:**
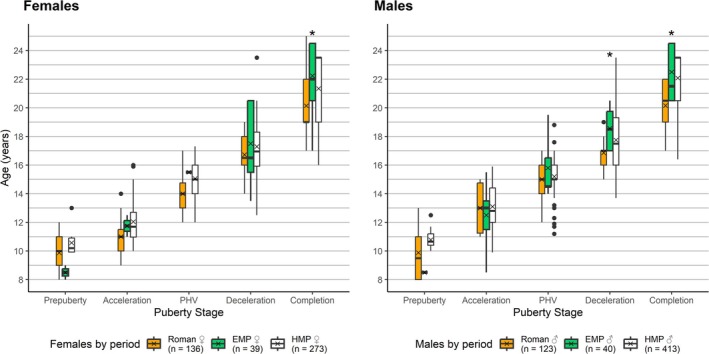
Age at puberty stage by sex in the Roman, early, and high medieval period. Dots signify outliers, asterisks mark statistically significant differences. Sex estimation in early medieval individuals (EMP in green) based on genetic sex. HMP (high/late medieval, in white), Roman (in orange).

When comparing the early medieval individuals from Austria with adolescents from Roman Imperial France and Italy, and late medieval England in a sex‐pooled sample, the early medieval adolescents consistently exhibited a higher mean age of stage attainment from the acceleration phase onwards (Figure 7). The only exception was during deceleration, where the late medieval individuals were generally older than the early medieval individuals of both sexes (Table 9, Figure 7). When observing the sex‐specific ages per puberty stage, late medieval individuals were older than early medieval individuals during the onset phase, and late medieval females also have the highest mean age during acceleration phase followed by early medieval females (Table 9, Figure 8). From PHV onwards early medieval individuals are consistently older than late medieval adolescents, apart from the separated deceleration stage phase, as mentioned above. In males, this pattern begins earlier, from the acceleration phase. Roman individuals were the youngest across all developmental stages. The Kruskalis‐Wallis test showed that this was statistically significant for males during the combined deceleration‐maturation (*p* = 0.04, $\varepsilon^{2}$ = 0.059) and completion phase (*p* < 0.001, *ε* ^2^ = 0.118) and for females in the completion stage (*p* < 0.001, *ε* ^2^ = 0.198) (Table 9, Figure 8).

The age of females in the pre‐menarche category was significantly different with a moderate effect when all Roman, early and late medieval females were compared (*p* < 0.01) (Table 10). Pre‐menarcheal age was lower in the Roman females compared to the medieval period, with the early medieval individuals (*n* = 4) having a slightly higher mean age compared to the later medieval, based on the available data. The post‐menarcheal ages were more similar, differing by less than 1 year. There is a steady change to higher ages in the oldest observed females in the pre‐menarcheal group from 14 years (Roman) to 15.5 years (early medieval) to 17.3 years (late medieval). Similarly, the age of the youngest females who achieved menarche in each period changed from 12 to 13.5 years to 15 years respectively.

**TABLE 10:** Age‐at‐menarche status between the Roman, early (EMP), and late medieval periods (LMP) of females aged 10–18 years.

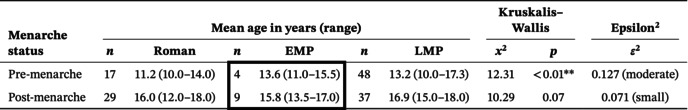

There are slight differences in the recording of puberty stages between the studies. We differentiated between prepuberty and onset stage by the mineralization stage of the mandibular canine (Falys and Lewis 2020). Prepuberty in previous studies equates to onset phase in this study. Further, the deceleration stage in Avery et al. (2023) combines features from deceleration and maturation stages used in this study and Lewis et al. (2016a, 2016b). Since all seven development stages were used in this study (Table 11), we combined deceleration and maturation stages for comparisons between the Roman and other individuals. One individual, classified as between the stages of deceleration and maturation, was included in the combined deceleration group because they fit Avery et al.'s (2023) deceleration stage. Additionally, we only compared individuals up to the age of 25 years with data from previous studies so as not to introduce age biases of sample selection into the statistical comparison.

**TABLE 11:** Differences in puberty stages nomenclature across studies compared in this study.

This study	Avery et al. 2023	Lewis et al. (2016b)
Pre‐puberty		
Onset/initiation	Pre‐puberty	Pre‐puberty
Acceleration	Acceleration	Acceleration
PHV	PHV	PHV
Deceleration	Dec‐Mat	Deceleration
Maturation		Maturation
Completion	Completion	Completion

## 4 Discussion

### 4.1 Timing of Adolescence and Biosocial Implications During the Early Medieval Period

This study provides the first reconstruction of adolescent development in rural populations from Austria, which occupied two close‐by cemeteries for roughly 150 years during the Avar period. This study, therefore, adds Central Europe as a new study area and the early medieval period as a new period for studying past adolescence. Moreover, for the first time in a study on past adolescence, comprehensive aDNA testing allowed for more reliable and detailed observations of sex‐specific ages of development.

Skeletal markers indicate that the first outward signs of puberty appeared around the age of 10 years for males, and based on mandibular canines alone, an age of 10 years is also suggested for females. After this point, growth accelerated in girls around the age of 12 years and in boys around 2 years later. PHV of the pubertal growth spurt happened around the age of 15 years in males, which is when clear signs of transition, such as the breaking of the voice, become obvious, and adolescents display the fastest increases in growth. Females were in the deceleration phase from 14 to 17 years on average. A deeper voice and facial hair are often corollaries of the deceleration phase around 17–18 years. Females likely attained menarche around the age of 15 years or more broadly between 14 and 17 years if we take the fusion times in the pelvis and the average age for the deceleration stage into account, which is when menarche usually occurs. Females were on average 16–21 years and males were between 17 and 21 years old when they were in the maturation phase, the last phase of the pubertal growth spurt, when final adult stature was almost reached, and adolescents outwardly appeared essentially as young adults. Growth was complete in some females as young as 17 years and in males between 18 and 23 years but certainly by age 23 in both sexes.

If the mean ages for this Austrian skeletal sample reflect the average ages of the living population, we can estimate that adolescent development took around 10 years in males from the onset of puberty to the end of the sexual maturation stage. Due to a lack of females in the onset phase, we cannot estimate the same for them; however, both sexes typically spend the same duration in the pubertal growth spurt even though the timing differs (Lewis 2022). Females developed earlier than males. The age difference of up to 2 years (Table 9) aligns well with the average difference of 1.5–2 years, commonly observed in modern populations (Bogin and Smith 1996). This suggests that the methods used in this study reflect real developmental differences between the sexes. In this sample, the ages of some individuals from the same puberty stage could diverge by as much as 7.5 years (Table 9). This suggests a wide range of when adolescents entered the pubertal growth spurt and potentially of how long they took to fully mature. The larger age ranges may reflect differences in genetics, fetal programming, diet, health, and heterogeneous living conditions in the communities, which are known to lead to differing timing of development (Ham and DeWitte 2022). Nonetheless, individual variability in adolescent growth can be high in normal populations. For example, Hägg and Taranger (1982) found a variation of six years in onset, PHV, and growth termination in both male and female Swedish adolescents. The archeological material supports that the studied late Avar populations were socioeconomically quite homogenous (Daim et al. 2024; Wang et al. 2025).

Material evidence from the burials at Leobersdorf and Mödling suggests that physiological changes during adolescence were recognized socially. Wang et al. (2025) observed that belt buckles were more frequently found in female graves older than 14 years. This corresponds with the broad observations that with PHV and menarche around this age, clear signs of transition are visible in adolescents (Table 12). The physiological change seems to have been reflected in the material culture in different clothes and attire. The variability in the age at which individuals were interred with certain grave goods may reflect subtle cultural distinctions between communities or differences in familial traditions. Alternatively, varying ages could indicate that personal attire was shaped by individual developmental trajectories—both biological and social—rather than a culturally standardized chronological age (Perry 2005). Similarly, Doe et al. (2025) showed that physiological changes correlated with burial location in medieval adolescents from Spain.

**TABLE 12:** Transitions in the appearance of adolescence during the early medieval period in Austria based on osteological indicators.

Adolescent growth phase	Adolescent growth remaining^c^	Physiological changes		Average ages^a^	
		♀	♂	♀	♂
Pre‐puberty	100%	Adrenarche, body odor changes, pubarche^b^, ^f^		< 10	< 9
Onset	80%–100%	Ovaries enlarge^c^	Testis start enlarging^g^	—	8–11
Acceleration	65%–80%	Breast buds^c^	Testes enlarge^f^	10–12	12–14
PHV	25%–65%	Breast development^c^	Breaking of voice^e^ Genitalia enlarge^f^ Spermarche^b^	ca. 15	14–18
		Body hair^f^			
Deceleration	10%–25%	Menarche^c^ Beginning fertility Irregular cycles^c^	Voice deepens^e^ Facial hair	14–17	17–18
Maturation	5%–10%	Generally adult appearence^d^		16–21	17–22
		Subfecundity^b^	Increased musculature^b^		
Completion	0%–5%	Adult stature and secondary sex characteristics^c^		> 20	> 20
		Full fertility after 3–7 years after menarche	Full beard growth		

^a^ Average ages from this study (in years) are based on 1 SD from the mean and rounded to the next year.

^b^ Bogin (2020, 2011, 281).

^c^ Blom et al. (2021, 6), based on Lewis (2016) and Shapland and Shapland (2013, 2014).

^d^ Falys and Lewis (2020, 11).

^e^ Hägg and Taranger (1982).

^f^ Tanner (1962).

^g^ Doe et al. (2025).

Frequently, menstruation is viewed as an outward sign of maturity and the beginning of the transition to adulthood (Perry 2005). Theoretically, reproduction is possible from around the time of menarche. However, females undergo a period of reduced fertility with irregular cycles for the 2–5 year period after menarche until growth and uterine maturation are completed (Itriyeva 2022). Therefore, reproduction in the Avar Period in Austria was anatomically more likely to begin around 17–22 years. Males, on the contrary, are technically fertile years before growth is completed. However, it is rare for boys to reproduce during their adolescent development (Gluckman and Hanson 2006). Consistent with this, an interdisciplinary study on four Avar Period cemeteries in Hungary used genetic pedigree reconstruction from which they concluded that females entered their reproductive age at 18–20 years based on the youngest observed mothers, who were 18–22 years old. The youngest fathers were 24–29 years old (Gnecchi‐Ruscone et al. 2024). These findings align well with the anticipated begin of reproductive age in this study for the Vienna Basin during the Avar Period.

### 4.2 Comparison to Roman and Late Medieval Contexts

When comparing the pooled mean ages of both sexes at each adolescent growth stage of the early medieval Austrian adolescents with the Imperial Roman and late medieval populations, the early medieval individuals appear to be generally older (Figure 7). However, when the sexes are compared separately, a more complex picture emerges (Figure 8). The late medieval individuals are the oldest during the onset phase. The difference could be affected by the small number of early medieval individuals, with only four males in the onset phase. Still, the onset of the puberty growth spurt might have started at a similar age in the compared time periods, around age 10 (Avery et al. 2023; Lewis et al. 2016b), but then the timing diverged with the progression of the growth spurt. In the case of males from the acceleration phase and for both sexes from PHV onwards, the early medieval individuals show a delay in comparison to the Roman and the late medieval adolescents (Figure 8). This is especially true for males in the later stages of deceleration–maturation and completion. Sex‐specific observations indicate that adolescent development happened later during the early medieval group or was potentially more stretched compared to the Roman and late medieval populations. Nevertheless, the timing could have developed differently in the sexes during the medieval periods since late medieval females were older during the deceleration phase, and early and late medieval males had a similar age in deceleration. Thus, early medieval puberty timing might not necessarily be delayed relative to the later medieval group in all of the phases of adolescence.

In general, the overall estimated average ages at menarche are similar for the continental Roman period (13–17 years), the early (14–17 years), and the late medieval period (15–17 years) (Avery et al. 2023; Lewis et al. 2016b). Fusing distal phalanges, considered a useful proxy for the time of menarche, was found in one early medieval female with a mean age of 15.5 years. Correspondingly, Lewis et al. (2016b) report this indicator in late medieval females from around 15 years, and based on supplemental data from Avery et al. (2023), the mean age of Roman females around menarche was around 15.1 years. Nevertheless, observations about the oldest and youngest individuals among pre‐ and post‐menarcheal females suggest that the age at menarche might have been lower in Roman and later in late medieval females compared to the early medieval females (Table 10).

In contrast to the hypothesis of earlier adolescent development in the early medieval period, the available osteological data indicate that early medieval adolescents from Austria were delayed compared to the populations from previous and following periods. Below, we will discuss what factors could have influenced the developmental timing during the early medieval period in Austria.

### 4.3 Determinants in Pubertal Timing Variation

Based on large‐scale comparisons of multiple palaeopathological indicators, health in Europe was more favorable during the early compared to the late medieval and to the Roman Imperial periods. This likely correlates to the absence of urbanization during this time (Steckel et al. 2018). In fact, population density is a key differentiator between the compared groups. The individuals from the Roman and the late medieval period come from cemeteries associated with small to large urban centres (Avery et al. 2023; Lewis et al. 2016b), whereas the early medieval settlements in Austria can be expected to be rural and of low population density (Pohl 2018). While not universal, bioarcheological studies largely support the urban penalty hypothesis, which finds worse health in urban communities compared to rural settlements (e.g., Betsinger and DeWitte 2021; Roberts 2009). Correspondingly, Lewis et al. (2016a) found late‐developing males at PHV in medieval London, suggesting that high urban stressors could have affected these adolescents. In addition, they found a link between tuberculosis and other infections with delayed PHV in late medieval adolescents (Lewis et al. 2016a). The older age of late medieval females during the deceleration stage may be linked to palaeopathological evidence showing that urban females carried the burden of respiratory disease in the late medieval period in England. Urban females also showed a higher prevalence of specific infections, such as tuberculosis and treponemal disease than their rural peers (Lewis 2016). Consequently, one could expect earlier development during the more rural early medieval period. On the contrary, early medieval adolescents showed higher mean ages during most growth stages.

Conversely, negative intra‐uterine conditions can also lead to earlier puberty (Gluckman and Hanson 2006). For example, individuals who were small for gestational age tend to show earlier adrenarche, pubarche, and age at menarche (Petraitiene et al. 2020). Potentially, mothers living in the urban groups suffered from the negative living environments during pregnancy, which could have affected their offspring to develop earlier during adolescence. Therefore, the observed later development in the Austrian sites could be due to the absence of urban centers in Central Europe during the early medieval period, which led to less stressful living environments during pregnancy.

Diachronic observations of femoral length and enamel hypoplasia also show that the urban penalty is not constant and is more severe in the Roman and early medieval periods, while late medieval urban dwellers might have fared better, judging by the lower prevalence of enamel hypoplasia (Bereczki et al. 2018; Meinzer et al. 2018). Additionally, while there is an observable trend toward more prevalent hypoplasia in large centers, Bereczki et al. (2018) and Meinzer et al. (2018) have hypothesized that smaller and medium‐sized towns might have benefited from trade networks, which contributed to protein‐rich and varied diets, buffering against regional droughts and famines. For the Roman period, social inequality likely played a major role in health outcomes (e.g., Redfern 2018). The predominantly middle‐class communities from the Roman period group might have lived in relatively advantageous conditions. Additionally, the medieval apprenticeship system, which brought rural adolescents to the towns, might further complicate a rural–urban comparison of adolescent health in the medieval period (Lewis 2016). Finally, the example of delayed development of a post‐medieval rural Dutch community linked to disease and famine (Blom et al. 2021) also highlights the need to carefully consider context‐specific living conditions when comparing rural and urban health outcomes (Betsinger and DeWitte 2021).

Differences in the genetic background could also explain differences in puberty timing. Historical, archeological, and genetic studies show that the Avar empire was an ethnically diverse population that differed from the Roman Period and from the late medieval English groups (Gnecchi‐Ruscone et al. 2022; Pohl 2018). Furthermore, genetic analysis shows that the Avar population in Austria had varying degrees of Eurasian admixture (Wang et al. 2025), which might also have contributed to the inter‐ and intra‐group variation in pubertal timing observed in this study.

### 4.4 Methodological Challenges

The comparison between the Austrian early medieval cemeteries and the Roman and late medieval populations could be affected by sampling bias. The early medieval sample is comparatively small, which could have skewed the results. Thus, the observed differences with the other populations in the puberty stages and between the sexes within the early medieval group should be cautiously interpreted. Bioarcheological studies on past adolescence have limitations in regard to the representativeness of the reconstructed ranges of development (Avery and Lewis 2023; Doe et al. 2019; Lewis 2022). Foremost, the mortality bias of the studied adolescents might not reflect the average development of the living population (Spake et al. 2022). To ameliorate this, we followed the recommendations from previous research to only compare reconstructed developmental timing from skeletal remains (Avery and Lewis 2023). Previous bioarcheological research has highlighted the imprecision of osteological sex estimation in non‐adults and the potential to mitigate this through aDNA or minimally destructive amelogenin peptide analysis (Avery and Lewis 2023; Gowland et al. 2021; Stewart et al. 2017). To the best of our knowledge, the present study is the first to estimate biological sex via aDNA for all the analyzed individuals. Therefore, the sex estimations in this study are considered to be robust. This allows for a more reliable sex‐specific pubertal timing for the studied adolescents. Importantly, since previous studies relied mostly on morphological sex estimation of non‐adults, a proportion of these adolescents might have been miscategorised. The observed sex‐specific differences might, therefore, not solely reflect differences in development but differences in the accuracy of sex estimation.

Slight differences in assessing adolescent development stages and menarche between this study and previous ones could also affect the results. Similarly, every study used slightly different age categories and different age estimation methods, which cause comparability issues. This could have affected the comparisons between the later development stages in particular. This is due to differences in the ranges of age categories and because epiphyseal fusion and third molar development are expected to be more variable overall (Doe et al. 2019). Additionally, age estimations using AlQahtani et al. (2010) were shown to produce higher mean ages compared to Moorrees et al. (1963). Šešelj et al. (2019) also report that Moorrees et al. (1963) underestimated ages compared to their study.

The diachronic comparison of two early medieval cemeteries in Austria with the Roman cemeteries from France and Italy and the late medieval skeletons from the UK assumes that these remains are representative of the respective chronological periods. However, we cannot ignore that these local data might not be representative of other contemporaneous contexts. Even though the timing of adolescence of the Roman adolescents from France and Italy are comparable, Avery et al. (2023) noted differences to their Romano‐British peers (see Arthur et al. 2016), which suggests variability in adolescent development in the Roman provinces. In addition, different health trends after the end of the Roman Empire in the coastal and continental regions of Croatia, for example, suggest that we can also expect local variability in development during the subsequent early medieval period (Šlaus 2008). Therefore, the observed differences in pubertal timing could be due to many factors other than chronological distance and population density.

### 4.5 Future Research

Studies on consecutive time periods within a defined ecogeographic setting are needed to draw clearer conclusions about the development of pubertal timing throughout the past. Bioarcheological studies on adolescent development could add further evidence on the effects of biological stress on phases of life history that have eluded bioarcheological studies before now (Lewis 2022). Future research could explore whether intra‐uterine stress in past urban environments contributed to accelerated development. Intra‐uterine stress could affect the fetus to adapt to an anticipated harsh postnatal environment. This could lead to accelerated maturation as a life history strategy to adapt to increased environmental risks to be able to reproduce earlier (Ham and DeWitte 2022; Gluckman and Hanson 2006).

The present study shows that establishing sex‐specific timing of adolescence is paramount to compare different studies. Future studies should aim to compare adolescents with robust biomolecular sex estimation using the same age estimation methods and developmental assessments to ensure comparability.

## 5 Conclusion

This study reconstructed the timing of the adolescent growth spurt and sexual maturation in the early medieval period in Central Europe for the first time. It also represents one of the few rural populations and the first to use genetically sex‐estimated individuals. The hypothesis of earlier pubertal timing due to a healthier rural environment during the early medieval period could not be confirmed based on the available data. On the contrary, the comparison with previous research points to a delay in developmental timing compared to the previous Roman and, to a lesser degree, the late medieval period. The relative delay might be due to a different genetic background or different living conditions. However, differences in socioeconomic status, methods, and sample size could have affected the results. In accordance with modern data, females were found to develop earlier than males. Females and males were fully adult by age 23, with females completing growth as young as 17 years. The average age of menarche was 14–17 years. There is evidence within the material culture that physiological changes were recognized and may relate to changing social roles as they approached adulthood. This study also highlights how comparisons between datasets on adolescence are affected by differences in puberty stage assessments and age estimation. Further standardization of these two aspects would enhance comparability. Additional research from different contexts, geographic regions, and time periods is needed to draw clearer conclusions about the development of pubertal timing in the past.

## Conflicts of Interest

The authors declare no conflicts of interest.

## Supporting information


**Table S1:** ajpa70123‐sup‐0001‐Supplement1.docx.

**Table S2:** ajpa70123‐sup‐0002‐Supplement2.docx.

**Table S3:** ajpa70123‐sup‐0003‐Supplement3.docx.

## Data Availability

The data that support the findings of this study are available in the Supporting Information of this article. To facilitate future comparisons using different age estimation methods, we included the dental scores of the early medieval individuals from this study (Tables S2 and S3).
