# Supplementary material for: Bioarcheological Perspectives on the Timing of Adolescence in Rural Avar‐Age Austria, 7th–9th Centuries ce
Source: Am J Biol Anthropol. 2025 Sep 17;188(1):e70123. doi: 10.1002/ajpa.70123 (PMC12441998; doi:10.1002/ajpa.70123)
Supplement: Supplementary file 1 — Table S1: ajpa70123‐sup‐0001‐Supplement1.docx. [file AJPA-188-e70123-s001.docx]

Supplement 1 Age categories and criteria.

| Age category | Feature | Fusion times (in years) | Reference |
| --- | --- | --- | --- |
| **8.0-17.9** | Mineralisation of permanent dentition | Mean ages | (AlQahtani et al., 2010) |
| **18.0-23.9** | Extended epiphysis of ischio-pubic ramus | Extends and fuses 18-21 | (Scheuer & Black, 2000) |
|  | Fusing or fused ischial epiphysis | 16-20 | (Scheuer & Black, 2000) |
|  | Unfused or flaked epiphysis at medial clavicle | Unfused/flaked: 18-21, fusing 18-23 | (Cardoso, 2008; Scheuer & Black, 2000) |
|  | Fusing scapular borders | 19-23 | (Cunningham et al., 2016) |
|  | Vertebral annual rings fusing | Fused: 18 ≤, fusing 18-23 | (Albert & McCallister, 2004; Cardoso & Rios, 2011; Scheuer & Black, 2000) |
|  | Unfused/fusing rib heads | Unfused: ≤19, fusing: 17-25, fused ≥17 | (Rios & Cardoso, 2009) |
|  | Gap between  sacral body S1-2 | 20-25 | (Cunningham et al., 2016) |
|  | Fusing acromial process | 18-20 | (Cunningham et al., 2016) |
|  | Anterior inferior iliac spine | 16-20 | (Cunningham et al., 2016) |
|  | Unfused jugular growth plate | 22-34 (H&H, 1997) 20-50; 75% open <35 (M&M, 1995) | (Hershkovitz et al., 1997; Maat & Mastwijk, 1995) |
|  | M3 until full mineralisation | Up to age 23 | (AlQahtani et al., 2010) |
| **24.0-25.9** | Ischio-pubis ramus fully fused | 18-21 | (Scheuer & Black, 2000) |
|  | Complete fusion of vertebral rings | Fused: 18 ≤, fusing 18-23 | (Albert & McCallister, 2004; Cardoso & Rios, 2011; Scheuer & Black, 2000) |
|  | Complete fusion of ischial epiphysis | Fuses 16-20 | (Cunningham et al., 2016) |
|  | Fused scapular borders | 19-23 | (Cunningham et al., 2016) |
|  | Gap between  sacral body S1-2 | 20-25  (closed 25+, open: <27) | (Cunningham et al., 2016) |
|  | Fusing medial clavicle epiphysis | 21-25 | (Cardoso, 2008; Scheuer & Black, 2000) |
|  | Fusing rib heads | Unfused: ≤19, fusing: 17-25, fused ≥17 | (Rios & Cardoso, 2009) |
|  | Unfused/fusing jugular growth plate | 22-34 (H&H, 1997) 20-50; 75% chance if open: <35 (M&M, 1995) | (Hershkovitz et al., 1997; Maat & Mastwijk, 1995) |
|  | Closed junction of sacral bodies S1-S2 | 20-25 (closed 25+, open: <27) | (Cunningham et al., 2016) |
| **26.0-30.9** | Fused medial clavicle | 20-25; (fusion incomplete <30) | (Cardoso, 2008; Scheuer & Black, 2000) |
|  | Fused rib heads | Unfused: ≤19, fusing: 17-25, fused ≥17 | (Rios & Cardoso, 2009) |
|  | Fusing jugular growth plate | 22-34 (H&H, 1997) 20-50; 75% chance if open: <35 (M&M, 1995) | (Hershkovitz et al., 1997; Maat & Mastwijk, 1995) |
|  | All epiphyses fused | 25+ |  |
| **30+** | Fused jugular growth plate | 22-34 (H&H, 1997) 20-50; 75% chance if open: <35 (M&M, 1995) | (Hershkovitz et al., 1997; Maat & Mastwijk, 1995) |
