# Supplementary material for: Bioarcheological Perspectives on the Timing of Adolescence in Rural Avar‐Age Austria, 7th–9th Centuries ce
Source: Am J Biol Anthropol. 2025 Sep 17;188(1):e70123. doi: 10.1002/ajpa.70123 (PMC12441998; doi:10.1002/ajpa.70123)
Supplement: Supplementary file 2 — Table S2: ajpa70123‐sup‐0002‐Supplement2.docx. [file AJPA-188-e70123-s003.docx]

Supplement 2 Individual data of early medieval individuals.

| **Stable identifier** NHMW-Anthro-OSTE-… | **Grave no.** | **Site** | **Mean age** | **Biological sex** | **Pubertal stage** | **Menarche status** |
| --- | --- | --- | --- | --- | --- | --- |
| 27694 | 9 | Mödling | 19.5 | female | Maturation | post-menarche |
| 27698 | 13 | Mödling | 18.5 | male | Maturation |  |
| 27699 | 15 | Mödling | 28 | male | Completion |  |
| 27718 | 33 | Mödling | 11 | female | Acceleration | pre-menarche |
| 27722 | 36.II | Mödling | 8.5 | female | Prepuberty | pre-menarche |
| 27730 | 44.I | Mödling | 24.5 | male | Completion |  |
| 27731 | 44.II | Mödling | 19.5 | female |  | post-menarche |
| 27735 | 47 | Mödling | 13 | male | Acceleration |  |
| 27737 | 51 | Mödling | 24.5 | female | Completion | post-menarche |
| 27740 | 54 | Mödling | 18.5 | female |  | post-menarche |
| 27741 | 55.I | Mödling | 15.5 | male | Acceleration |  |
| 27744 | 57 | Mödling | 13 | male | Acceleration |  |
| 27760 | 80 | Mödling | 8.5 | male | Prepuberty |  |
| 27764 | 81D | Mödling | 19.5 | female |  | post-menarche |
| 27771 | 88 | Mödling | 20.5 | female | Completion | post-menarche |
| 27774 | 92 | Mödling | 24.5 | female | Completion | post-menarche |
| 27778 | 96 | Mödling | 17 | female | Deceleration | post-menarche |
| 27791 | 111 | Mödling | 28 | male | Completion |  |
| 27800 | 122 | Mödling | 28 | male | Completion |  |
| 27806 | 128 | Mödling | 20.5 | female | Completion | post-menarche |
| 27811 | 133 | Mödling | 28 | male | Completion |  |
| 27818 | 144 | Mödling | 20.5 | female | Completion | post-menarche |
| 27827 | 153 | Mödling | 8 | female | Prepuberty | pre-menarche |
| 27835 | 161 | Mödling | 14.5 | male | PHV |  |
| 27839 | 167A | Mödling | 17 | female | Completion | post-menarche |
| 27843 | 169 | Mödling | 12 | male | Onset |  |
| 27845 | 171 | Mödling | 18.5 | female |  | post-menarche |
| 27860 | 187 | Mödling | 24.5 | female | Completion | post-menarche |
| 27861 | 188 | Mödling | 28 | male | Completion |  |
| 27866 | 192 | Mödling | 14 | male | PHV |  |
| 27869 | 197 | Mödling | 22 | female | Completion | post-menarche |
| 27878 | 206 | Mödling | 20.5 | male | Completion |  |
| 27881 | 209 | Mödling | 20.5 | female |  | post-menarche |
| 27886 | 214 | Mödling | 18.5 | female |  | post-menarche |
| 27900 | 236 | Mödling | 8.5 | male | Prepuberty |  |
| 27914 | 254 | Mödling | 11.5 | female |  | pre-menarche |
| 27916 | 256A | Mödling | 21.5 | male | Completion |  |
| 27917 | 256B | Mödling | 8.5 | male | Prepuberty |  |
| 27923 | 263 | Mödling | 20.5 | male | Maturation |  |
| 27925 | 265A | Mödling | 24.5 | female | Completion | post-menarche |
| 27936 | 275 | Mödling | 20.5 | female | Maturation | post-menarche |
| 27946 | 285 | Mödling | 19.5 | female | Completion | post-menarche |
| 27960 | 298 | Mödling | 14 | female |  | pre-menarche |
| 27971 | 309 | Mödling | 20.5 | female | Completion | post-menarche |
| 27978 | 315B | Mödling | 14.5 | male | Acceleration |  |
| 27984 | 321 | Mödling | 28 | female | Completion | post-menarche |
| 28003 | 340 | Mödling | 10 | female |  | pre-menarche |
| 28008 | 345 | Mödling | 24.5 | female | Completion | post-menarche |
| 28014 | 352B | Mödling | 10.5 | male | Onset |  |
| 28030 | 369 | Mödling | 15.5 | female |  | pre-menarche? |
| 28044 | 382B | Mödling | 21.5 | male | Completion |  |
| 28046 | 384 | Mödling | 18.5 | male | Deceleration |  |
| 28048 | 386 | Mödling | 16.5 | male | PHV |  |
| 28050 | 388 | Mödling | 13 | male | Acceleration |  |
| 28054 | 389D | Mödling | 10 | male | Onset |  |
| 28055 | 390 | Mödling | 19.5 | male | PHV |  |
| 28056 | 391 | Mödling | 13 | female |  | pre-menarche |
| 28059 | 394 | Mödling | 24.5 | male | Completion |  |
| 28073 | 408 | Mödling | 15.5 | female | PHV | pre-menarche |
| 28074 | 409 | Mödling | 16 | female | Maturation | post-menarche |
| 28079 | 415 | Mödling | 17 | male | Deceleration |  |
| 28081 | 418 | Mödling | 24.5 | male | Completion |  |
| 28084 | 422 | Mödling | 20.5 | male | Completion |  |
| 28088 | 426 | Mödling | 17 | female | Deceleration | post-menarche |
| 28116 | 456 | Mödling | 8.5 | male | Prepuberty |  |
| 28121 | 461 | Mödling | 12.5 | female | Acceleration | pre-menarche |
| 28126 | 466 | Mödling | 19.5 | male | Dec-Mat |  |
| 28163 | 505A | Mödling | 28 | male | Completion |  |
| 28168 | 508 | Mödling | 24.5 | female | Completion | post-menarche |
| 28172 | 512 | Mödling | 9 | female | Prepuberty | pre-menarche |
| 28185 | 526 | Mödling | 15.5 | female | Deceleration | post-menarche |
| 28186 | 527 | Mödling | 20.5 | male | Maturation |  |
| 28197 | 540 | Mödling | 17 | male | Deceleration |  |
| 28200 | 545 | Mödling | 9 | female |  | pre-menarche |
| 1003314 | 6 | Leobersdorf | 15.5 | female |  | post-menarche |
| 1003321 | 17 | Leobersdorf | 20.5 | female |  | post-menarche |
| 1003322 | 18 | Leobersdorf | 20.5 | female | Completion | post-menarche |
| 1003323 | 19 | Leobersdorf | 15.5 | female | Maturation | post-menarche |
| 1003329 | 25 | Leobersdorf | 16 | female | Maturation | post-menarche |
| 1003331 | 28 | Leobersdorf | 24.5 | female | Completion | post-menarche |
| 1003332 | 30 | Leobersdorf | 20.5 | female | Maturation | post-menarche |
| 1003334 | 34 | Leobersdorf | 20.5 | female | Completion | post-menarche |
| 1003345 | 45 | Leobersdorf | 20.5 | female | Maturation | post-menarche |
| 1003362 | 66 | Leobersdorf | 20.5 | male | Completion |  |
| 1003363 | 67 | Leobersdorf | 20.5 | female | Completion | post-menarche |
| 1003364 | 68 | Leobersdorf | 11.5 | female |  | pre-menarche |
| 1003371 | 76 | Leobersdorf | 13.5 | female | Deceleration | post-menarche |
| 1003376 | 81 | Leobersdorf | 24.5 | male | Completion |  |
| 1003377 | 82 | Leobersdorf | 24.5 | female | Completion | post-menarche |
| 1003387 | 103B | Leobersdorf | 13.5 | male | Acceleration |  |
| 1003388 | 104 | Leobersdorf | 28 | female | Completion | post-menarche |
| 1003396 | 114 | Leobersdorf | 24.5 | female | Completion | post-menarche |
| 1003397 | 116 | Leobersdorf | 20.5 | male | Completion |  |
| 1003399 | 119 | Leobersdorf | 24.5 | male | Completion |  |
| 1003400 | 120 | Leobersdorf | 24.5 | male | Completion |  |
| 1003412 | 134 | Leobersdorf | 8.5 | male | Onset |  |
| 1003413 | 135 | Leobersdorf | 15.5 | female | PHV | pre-menarche |
| 1003414 | 136 | Leobersdorf | 20.5 | male | Completion |  |
| 1003417 | 141 | Leobersdorf | 12.5 | male | Acceleration |  |
| 1003422 | 147 | Leobersdorf | 14.5 | male | PHV |  |
| 1003423 | 148 | Leobersdorf | 15 | female | Deceleration | around menarche |
| 1003424 | 149 | Leobersdorf | 20.5 | female | Maturation | post-menarche |
| 1003427 | 152 | Leobersdorf | 17 | male | Deceleration |  |
