# Supplementary material for: Bioarcheological Perspectives on the Timing of Adolescence in Rural Avar‐Age Austria, 7th–9th Centuries ce
Source: Am J Biol Anthropol. 2025 Sep 17;188(1):e70123. doi: 10.1002/ajpa.70123 (PMC12441998; doi:10.1002/ajpa.70123)
Supplement: Supplementary file 3 — Table S3: ajpa70123‐sup‐0003‐Supplement3.docx. [file AJPA-188-e70123-s002.docx]

Supplement 3 Dental scores from Mödling and Leobersdorf.

| Stable identifier NHMW-Anthro-OSTE-… | Site | Grave no. | Biological sex | Skeletal age | **UM3** | **UM2** | **UM1** | **UPM4** | **UPM3** | **UC** | **UI2** | **UI1** | **LM3** | **LM2** | **LM1** | **LPM4** | **LPM3** | **LC** | **LI2** | **LI1** |
| --- | --- | --- | --- | --- | --- | --- | --- | --- | --- | --- | --- | --- | --- | --- | --- | --- | --- | --- | --- | --- |
| 27694 | Mödling | 9 | female |  |  |  |  |  |  |  |  |  | Rc |  |  |  |  |  |  |  |
| 27698 | Mödling | 13 | male |  | R3/4 |  |  |  |  |  |  |  |  |  |  |  |  |  |  |  |
| 27699 | Mödling | 15 | male | 26-30 |  |  |  |  |  |  |  |  |  |  |  |  |  |  |  |  |
| 27718 | Mödling | 33 | female |  | Crc | R1/2 | R3/4 |  | R1/2 | A1/2 |  |  | Cr3/4 | R1/2 |  |  |  | A1/2 |  |  |
| 27722 | Mödling | 36.II | female |  |  |  | R1/2 |  |  | R1/4 | R1/2 | R1/2 |  | Crc-Ri |  |  | R1/4 | R1/4 | R3/4 | R3/4 |
| 27730 | Mödling | 44.I | male | 24-25 |  |  |  |  |  |  |  |  |  |  |  |  |  |  |  |  |
| 27731 | Mödling | 44.II | female |  | Rc |  |  |  |  |  |  |  | Rc |  |  |  |  |  |  |  |
| 27735 | Mödling | 47 | male |  |  |  |  | Rc | Rc-A1/2 | Rc |  |  |  | R3/4 |  | R3/4 | Rc-A1/2 |  |  |  |
| 27737 | Mödling | 51 | female | 24-25 |  |  |  |  |  |  |  |  |  |  |  |  |  |  |  |  |
| 27740 | Mödling | 54 | female |  |  |  |  |  |  |  |  |  | R3/4 |  |  |  |  |  |  |  |
| 27741 | Mödling | 55.I | male |  |  |  |  |  |  |  |  |  |  | A1/2 |  |  |  |  |  |  |
| 27744 | Mödling | 57 | male |  |  | R3/4 |  |  |  |  |  |  |  | R3/4 |  |  |  |  |  |  |
| 27760 | Mödling | 80 | male |  |  | Ri | R3/4 |  | R1/4 | R1/4 |  |  |  | Ri | R3/4 |  |  | Ri | R1/2 | R1/2 |
| 27764 | Mödling | 81D | female |  | Rc |  |  |  |  |  |  |  |  |  |  |  |  |  |  |  |
| 27771 | Mödling | 88 | female | 18-23 |  |  |  |  |  |  |  |  |  |  |  |  |  |  |  |  |
| 27774 | Mödling | 92 | female | 24-25 |  |  |  |  |  |  |  |  |  |  |  |  |  |  |  |  |
| 27778 | Mödling | 96 | female |  | R1/2 |  |  |  |  |  |  |  | R1/2 |  |  |  |  |  |  |  |
| 27791 | Mödling | 111 | male | 26-30 |  |  |  |  |  |  |  |  |  |  |  |  |  |  |  |  |
| 27800 | Mödling | 122 | male | 26-30 |  |  |  |  |  |  |  |  |  |  |  |  |  |  |  |  |
| 27806 | Mödling | 128 | female | 18-23 |  |  |  |  |  |  |  |  |  |  |  |  |  |  |  |  |
| 27811 | Mödling | 133 | male | 26-30 |  |  |  |  |  |  |  |  |  |  |  |  |  |  |  |  |
|  | Mödling | 144 | female | 18-23 |  |  |  |  |  |  |  |  |  |  |  |  |  |  |  |  |
| 27827 | Mödling | 153 | female |  |  | Ri |  | Ri |  | R1/4 |  | R3/4 |  | Crc | R3/4 | Ri | Ri | Ri-R1/4 | R1/2 |  |
| 27835 | Mödling | 161 | male |  | R1/4 |  |  |  |  |  |  |  | Ri |  |  |  |  |  |  |  |
| 27839 | Mödling | 167A | female |  |  |  |  |  |  |  |  |  | R1/2 |  |  |  |  |  |  |  |
| 27843 | Mödling | 169 | male |  | Cr3/4-Ri |  |  |  | R1/2 |  |  | A1/2 |  |  |  |  |  | R1/2 |  |  |
| 27845 | Mödling | 171 | female |  | R3/4 |  |  |  |  |  |  |  | R3/4 |  |  |  |  |  |  |  |
| 27860 | Mödling | 187 | female | 24-25 |  |  |  |  |  |  |  |  |  |  |  |  |  |  |  |  |
| 27861 | Mödling | 188 | male | 26-30 |  |  |  |  |  |  |  |  |  |  |  |  |  |  |  |  |
| 27866 | Mödling | 192 | male |  | R1/4 | R3/4 |  |  |  |  |  |  | R1/4 | R3/4 |  |  |  |  |  |  |
| 27869 | Mödling | 197 | female |  |  |  |  |  |  |  |  |  | A1/2 |  |  |  |  |  |  |  |
| 27878 | Mödling | 206 | male | 18-23 |  |  |  |  |  |  |  |  |  |  |  |  |  |  |  |  |
| 27881 | Mödling | 209 | female | 18-23 |  |  |  |  |  |  |  |  |  |  |  |  |  |  |  |  |
| 27886 | Mödling | 214 | female |  |  |  |  |  |  |  |  |  | R3/4 |  |  |  |  |  |  |  |
| 27900 | Mödling | 236 | male |  |  | Ri |  |  |  | Ri |  |  |  | Ri | R3/4 |  |  | Ri | R1/2 |  |
| 27913 | Mödling | 254 | female |  |  | R1/2 | A1/2 | R3/4 | R3/4 | R3/4 | A1/2- | A1/2 | Cr3/4 | R3/4 |  | R3/4 | R3/4 | Rc |  |  |
| 27916 | Mödling | 256A | male |  |  |  |  |  |  |  |  |  | A1/2 |  |  |  |  |  |  |  |
| 27917 | Mödling | 256B | male |  |  | Ri | R3/4 | Ri | Ri | R1/4 | R1/4 | R1/2 |  | Ri-R1/4 | R3/4 | R1/4 | R1/4 | R1/4 | R3/4 | Rc |
| 27923 | Mödling | 263 | male | 18-23 |  |  |  |  |  |  |  |  |  |  |  |  |  |  |  |  |
| 27924 | Mödling | 264 | male | 24-25 | R3/4 |  |  |  |  |  |  |  | R3/4 |  |  |  |  |  |  |  |
| 27925 | Mödling | 265A | female | 18-23 |  |  |  |  |  |  |  |  |  |  |  |  |  |  |  |  |
| 27936 | Mödling | 275 | female | 18-23 |  |  |  |  |  |  |  |  |  |  |  |  |  |  |  |  |
| 27946 | Mödling | 285 | female |  | Rc-A1/2 |  |  |  |  |  |  |  |  |  |  |  |  |  |  |  |
| 27960 | Mödling | 298 | female | 18-23 | Ri |  |  |  |  |  |  |  | Ri | Rc-A1/2 |  |  |  |  |  |  |
| 27962 | Mödling | 300 | male |  | Ri | R3/4 |  | Rc |  |  |  |  | Ri | R3/4 |  | Rc |  | Rc |  |  |
| 27971 | Mödling | 309 | female | 26-30 |  |  |  |  |  |  |  |  |  |  |  |  |  |  |  |  |
| 27978 | Mödling | 315B | male |  | R1/4 |  |  | A1/2 | Rc | A1/2 |  |  | Ri | Rc |  |  |  |  |  |  |
| 27984 | Mödling | 321 | female | 24-25 |  |  |  |  |  |  |  |  |  |  |  |  |  |  |  |  |
| 28003 | Mödling | 340 | female |  |  |  |  |  | R1/4 |  |  |  |  | R1/2 |  | R1/4 | R1/2 | R1/2 |  |  |
| 28008 | Mödling | 345 | female | 24-25 |  |  |  |  |  |  |  |  |  |  |  |  |  |  |  |  |
| 28014 | Mödling | 352B | male |  |  |  |  |  |  |  |  |  |  | R1/2 |  | R1/2 | R1/2 | R1/2 |  |  |
| 28030 | Mödling | 369 | female |  |  | A1/2 |  |  |  |  |  |  |  | Rc-A1/2 |  |  |  |  |  |  |
| 28044 | Mödling | 382B | male |  |  |  |  |  |  |  |  |  | A1/2 |  |  |  |  |  |  |  |
| 28046 | Mödling | 384 | male |  | R3/4 |  |  |  |  |  |  |  |  |  |  |  |  |  |  |  |
| 28048 | Mödling | 386 | male |  | R1/2 |  |  |  |  |  |  |  | R1/2 |  |  |  |  |  |  |  |
| 28050 | Mödling | 388 | male | 19.5 | Ri | R3/4-Rc |  |  |  |  |  |  |  | R1/2-3/4 |  |  |  |  |  |  |
| 28054 | Mödling | 389D | male |  |  | R1/4 | A1/2 |  |  |  |  |  |  | R1/2 | Rc | R1/2 | R1/2 | R1/2 |  |  |
| 28055 | Mödling | 390 | male | 24-25 |  |  |  |  |  |  |  |  | Rc |  |  |  |  |  |  |  |
| 28056 | Mödling | 391 | female |  | Ri-R1/4 | R3/4 |  | R3/4 | R3/4 | R3/4 |  |  | Ri | R3/4 |  | R3/4 | Rc | A1/2 |  |  |
| 28059 | Mödling | 394 | male | 24-25 |  |  |  |  |  |  |  |  |  |  |  |  |  |  |  |  |
| 28073 | Mödling | 408 | Female- |  | Ri-R1/4 | A1/2 |  |  |  |  |  |  | Ri |  |  |  |  |  |  |  |
| 28074 | Mödling | 409 | female | 24-25 |  |  |  |  |  |  |  |  | R3/4 | A1/2 |  |  |  |  |  |  |
| 28079 | Mödling | 415 | male | 18-23 | R1/2 |  |  |  |  |  |  |  | R1/2-3/4 |  |  |  |  |  |  |  |
| 28081 | Mödling | 418 | male | 24-25 |  |  |  |  |  |  |  |  |  |  |  |  |  |  |  |  |
| 28084 | Mödling | 422 | male | 18-23 |  |  |  |  |  |  |  |  |  |  |  |  |  |  |  |  |
| 28088 | Mödling | 426 | female |  | R1/2 |  |  |  |  |  |  |  |  |  |  |  |  |  |  |  |
| 28116 | Mödling | 456 | male |  |  | Ri |  |  |  |  |  |  |  | Ri | R3/4 | Ri | Ri | R1/4 |  |  |
| 28121 | Mödling | 461 | female | 26-30 |  | R3/4-Rc |  | R3/4-Ac | R3/4 |  |  |  |  | R3/4 |  |  |  |  |  |  |
| 28126 | Mödling | 466 | male | 24-25 |  |  |  |  |  |  |  |  | Rc |  |  |  |  |  |  |  |
| 28163 | Mödling | 505A | male | 26-30 |  |  |  |  |  |  |  |  |  |  |  |  |  |  |  |  |
| 28168 | Mödling | 508 | female | 24-25 |  |  |  |  |  |  |  |  |  |  |  |  |  |  |  |  |
| 28172 | Mödling | 512 | female |  |  |  |  |  |  |  |  |  |  |  | R1/4 | R1/4 |  | R1/4 | Rc |  |
| 28185 | Mödling | 526 | female |  | R1/4 | Rc-A1/2 |  |  |  |  |  |  |  | A1/2 |  |  |  |  |  |  |
| 28186 | Mödling | 527 | male | 18-23 |  |  |  |  |  |  |  |  |  |  |  |  |  |  |  |  |
| 28197 | Mödling | 540 | male |  | R1/2 |  |  |  |  |  |  |  | R1/2 |  |  |  |  |  |  |  |
| 28200 | Mödling | 545 | female | 18-23 |  | R1 |  |  | Ri-R1/4 | R1/4 | R1/2 | Rc |  | R1/4 | A1/2 |  |  | R1/4 |  | Rc |
| 1003314 | Leobersdorf | 6 | female | 18-23 | Ri |  |  |  |  |  |  |  |  |  |  |  |  |  |  |  |
| 1003321 | Leobersdorf | 17 | female | 18-23 |  |  |  |  |  |  |  |  |  |  |  |  |  |  |  |  |
| 1003322 | Leobersdorf | 18 | female | 18-23 |  |  |  |  |  |  |  |  |  |  |  |  |  |  |  |  |
| 1003323 | Leobersdorf | 19 | female | 24-25 | R1/4 |  |  |  |  | Rc |  |  |  | A1/2 |  |  |  |  |  |  |
| 1003329 | Leobersdorf | 25 | female | 18-23 |  |  |  |  |  |  |  |  | R1/4 |  |  |  |  |  |  |  |
| 1003331 | Leobersdorf | 28 | female | 18-23 |  |  |  |  |  |  |  |  |  |  |  |  |  |  |  |  |
| 1003332 | Leobersdorf | 30 | female | 18-23 |  |  |  |  |  |  |  |  |  |  |  |  |  |  |  |  |
| 1003334 | Leobersdorf | 34 | female | 18-23 |  |  |  |  |  |  |  |  |  |  |  |  |  |  |  |  |
| 1003345 | Leobersdorf | 45 | female | 18-23 |  |  |  |  |  |  |  |  |  |  |  |  |  |  |  |  |
| 1003362 | Leobersdorf | 66 | male | 18-23 |  |  |  |  |  |  |  |  |  |  |  |  |  |  |  |  |
| 1003363 | Leobersdorf | 67 | female | 18-23 |  |  |  |  |  |  |  |  |  |  |  |  |  |  |  |  |
| 1003364 | Leobersdorf | 68 | female | 24-25 |  |  |  | R3/4 |  | R3/4 |  |  |  | R1/2 |  |  |  |  |  |  |
| 1003371 | Leobersdorf | 76 | female | 24-25 |  | R3/4 |  |  |  |  |  |  |  | R1/4 |  |  |  |  |  |  |
| 1003376 | Leobersdorf | 81 | male | 24-25 |  |  |  |  |  |  |  |  |  |  |  |  |  |  |  |  |
| 1003377 | Leobersdorf | 82 | male | 26-30 | Ri | R3/4 |  | R3/4 | R3/4 | R3/4 |  |  | Ri | R3/4 |  | Rc |  |  |  |  |
| 1003387 | Leobersdorf | 82 | female | 24-25 |  |  |  |  |  |  |  |  |  |  |  |  |  |  |  |  |
| 1003388 | Leobersdorf | 103 | male | 18-23 |  | R3/4 |  |  | A1/2 |  |  |  |  | Rc |  |  |  |  |  |  |
| 1003396 | Leobersdorf | 114 | female | 24-25 |  |  |  |  |  |  |  |  |  |  |  |  |  |  |  |  |
| 1003397 | Leobersdorf | 116 | male | 24-25 |  |  |  |  |  |  |  |  |  |  |  |  |  |  |  |  |
| 1003399 | Leobersdorf | 119A | male | 24-25 |  |  |  |  |  |  |  |  |  |  |  |  |  |  |  |  |
| 1003400 | Leobersdorf | 120 | male | 24-25 |  |  |  |  |  |  |  |  |  |  |  |  |  |  |  |  |
| 1003412 | Leobersdorf | 134 | male | 18-23 |  | Ri | Rc |  |  |  |  | A1/2 |  |  | A1/2 |  |  | R3/4 |  |  |
| 1003413 | Leobersdorf | 135 | female |  | R1/4 |  |  |  |  |  |  |  | R1/4 |  |  |  |  |  |  |  |
| 1003414 | Leobersdorf | 136 | male | 18-23 |  |  |  |  |  |  |  |  |  |  |  |  |  |  |  |  |
| 1003417 | Leobersdorf | 141 | male |  | Crc | R3/4 |  | Rc | R3/4 | Rc |  |  |  | R3/4 |  | R3/4 |  | Rc |  |  |
| 1003422 | Leobersdorf | 147 | male | 18-23 | Ri-R1/4 |  |  |  |  |  |  |  | Ri |  |  |  |  |  |  |  |
| 1003423 | Leobersdorf | 148 | female |  | R1/4 |  |  |  |  |  |  |  | Ri |  |  |  |  |  |  |  |
| 1003424 | Leobersdorf | 149 | female | 18-23 |  |  |  |  |  |  |  |  |  |  |  |  |  |  |  |  |
| 1003427 | Leobersdorf | 152 | male |  | R1/2 |  |  |  |  |  |  |  | R1/2 |  |  |  |  |  |  |  |
